# (8*S*,9*R*,10*S*,11*S*,13*S*,14*S*,16*S*,17*R*)-4,4-Dichloro-16β-methyl-3,20-dioxo-17,21-bis­(propano­yloxy)-5β,8β-epoxy­pregna-1,9-diene

**DOI:** 10.1107/S1600536809028293

**Published:** 2009-07-22

**Authors:** Kamal Aziz Ketuly, A. Hamid A. Hadi, Seik Weng Ng

**Affiliations:** a Department of Chemistry, University of Malaya, 50603 Kuala Lumpur, Malaysia

## Abstract

The title compound, C_28_H_34_Cl_2_O_7_, is a derivative of the glucocorticoid steroid beclomethasone dipropionate. It features an expoxide linkage [angle at oxygen = 96.6 (2)°]. The dichlorocyclohexenone ring adopts an envelope conformation, with the C atom bearing the two Cl substituents representing the flap. The dichloro­methyl C atom deviates by 0.471 (4) Å from the plane defined by the other five atoms, whose maximum r.m.s. deviation is 0.04 Å.

## Related literature

For related structures, see: Ketuly *et al.* (2009*a* ▶,*b* ▶).
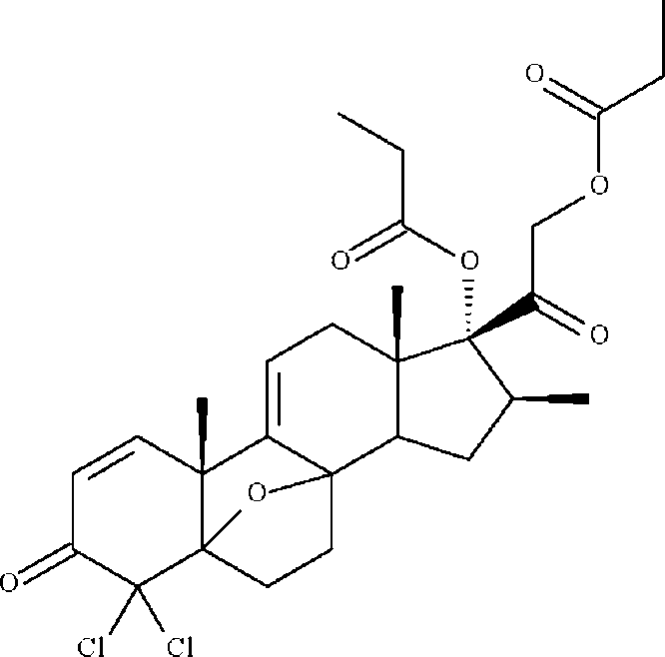


## Experimental

### 

#### Crystal data

C_28_H_34_Cl_2_O_7_
*M* *_r_* = 553.45
Orthorhombic, 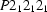

*a* = 11.1556 (2) Å
*b* = 12.2281 (2) Å
*c* = 19.3476 (4) Å
*V* = 2639.24 (8) Å^3^
*Z* = 4
Mo *K*α radiation
μ = 0.29 mm^−1^
*T* = 140 K
0.30 × 0.15 × 0.10 mm

#### Data collection

Bruker SMART APEX diffractometer
Absorption correction: multi-scan (*SADABS*; Sheldrick, 1996 ▶) *T*_min_ = 0.918, *T*_max_ = 0.971
24071 measured reflections
6067 independent reflections
4878 reflections with *I* > 2σ(*I*)
*R*_int_ = 0.083

#### Refinement

*R*[*F* ^2^ > 2σ(*F* ^2^)] = 0.048
*wR*(*F* ^2^) = 0.116
*S* = 1.03
6067 reflections
339 parameters
H-atom parameters constrained
Δρ_max_ = 0.45 e Å^−3^
Δρ_min_ = −0.29 e Å^−3^
Absolute structure: Flack (1983 ▶), 3199 Friedel pairs
Flack parameter: −0.16 (6)

### 

Data collection: *APEX2* (Bruker, 2008 ▶); cell refinement: *SAINT* (Bruker, 2008 ▶); data reduction: *SAINT*; program(s) used to solve structure: *SHELXS97* (Sheldrick, 2008 ▶); program(s) used to refine structure: *SHELXL97* (Sheldrick, 2008 ▶); molecular graphics: *X-SEED* (Barbour, 2001 ▶); software used to prepare material for publication: *publCIF* (Westrip, 2009 ▶).

## Supplementary Material

Crystal structure: contains datablocks global, I. DOI: 10.1107/S1600536809028293/bt5010sup1.cif

Structure factors: contains datablocks I. DOI: 10.1107/S1600536809028293/bt5010Isup2.hkl

Additional supplementary materials:  crystallographic information; 3D view; checkCIF report

## Figures and Tables

**Fig. 1.**
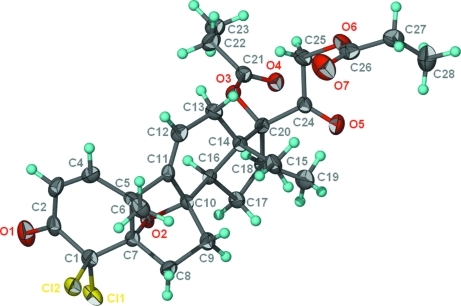
Anisotropic displacement ellipsoid plot (Barbour, 2001) of C28H34Cl2O7 at the 70% probability level. Hydrogen atoms are drawn as spheres of arbitrary radius.

## supplementary crystallographic information

### Experimental

The synthesis of the compound is described in a 1988 study commissioned by the Glaxo company (now called Glaxo Smith Kline), External Report No. WAP/88/007. Crystals were grown from its solution in ethyl acetate; m.p. 492–493 K. C7H elemental analysis. Calc. for C_28_H_34_Cl_2_O_7 _(MW 552): C 60.87, H, 6.15, Cl 14.26%. Found C 61.36, H 6.33, Cl 14.26 %.

### Refinement

Hydrogen atoms were placed at calculated positions (C–H 0.95–0.99 Å) and were treated as riding on their parent carbon atoms, with *U*(H) set to 1.2–1.5 times *U*_eq_(*C*).

### Crystal data

**Table:**

C_28_H_34_Cl_2_O_7_	*F*(000) = 1168
*M**_r_* = 553.45	*D*_x_ = 1.393 Mg m^−^^3^
Orthorhombic, *P*2_1_2_1_2_1_	Mo *K*α radiation, λ = 0.71073 Å
Hall symbol: P 2ac 2ab	Cell parameters from 6462 reflections
*a* = 11.1556 (2) Å	θ = 2.5–28.0°
*b* = 12.2281 (2) Å	µ = 0.29 mm^−^^1^
*c* = 19.3476 (4) Å	*T* = 140 K
*V* = 2639.24 (8) Å^3^	Multiple crystalline block, colorless
*Z* = 4	0.30 × 0.15 × 0.10 mm

### Data collection

**Table:**

Bruker SMART APEX diffractometer	6067 independent reflections
Radiation source: fine-focus sealed tube	4878 reflections with *I* > 2σ(*I*)
graphite	*R*_int_ = 0.083
ω scans	θ_max_ = 27.5°, θ_min_ = 2.0°
Absorption correction: multi-scan (*SADABS*; Sheldrick, 1996)	*h* = −14→14
*T*_min_ = 0.918, *T*_max_ = 0.971	*k* = −15→15
24071 measured reflections	*l* = −24→25

### Refinement

**Table:**

Refinement on *F*^2^	Secondary atom site location: difference Fourier map
Least-squares matrix: full	Hydrogen site location: inferred from neighbouring sites
*R*[*F*^2^ > 2σ(*F*^2^)] = 0.048	H-atom parameters constrained
*wR*(*F*^2^) = 0.116	*w* = 1/[σ^2^(*F*_o_^2^) + (0.0588*P*)^2^] where *P* = (*F*_o_^2^ + 2*F*_c_^2^)/3
*S* = 1.03	(Δ/σ)_max_ = 0.001
6067 reflections	Δρ_max_ = 0.45 e Å^−^^3^
339 parameters	Δρ_min_ = −0.29 e Å^−^^3^
0 restraints	Absolute structure: Flack (1983), 3199 Friedel pairs
Primary atom site location: structure-invariant direct methods	Flack parameter: −0.16 (6)

### Fractional atomic coordinates and isotropic or equivalent isotropic displacement parameters (Å^2^)

**Table:**

	*x*	*y*	*z*	*U*_iso_*/*U*_eq_	
Cl1	0.59256 (6)	0.63874 (6)	0.83223 (4)	0.03035 (17)	
Cl2	0.40010 (7)	0.78271 (6)	0.78724 (3)	0.02583 (16)	
O1	0.5154 (2)	0.76634 (19)	0.94879 (11)	0.0395 (6)	
O2	0.39813 (17)	0.49261 (14)	0.87881 (9)	0.0186 (4)	
O3	0.23417 (17)	0.17101 (16)	1.02004 (9)	0.0207 (4)	
O4	0.30538 (19)	0.00381 (17)	1.04842 (10)	0.0270 (5)	
O5	0.1490 (2)	−0.00893 (18)	0.88479 (11)	0.0311 (5)	
O6	−0.04283 (18)	−0.03711 (18)	0.96500 (10)	0.0289 (5)	
O7	−0.1240 (2)	0.0925 (2)	0.89835 (13)	0.0441 (6)	
C1	0.4397 (2)	0.6766 (2)	0.84723 (14)	0.0217 (6)	
C2	0.4287 (3)	0.7295 (2)	0.91964 (14)	0.0247 (6)	
C3	0.3070 (3)	0.7333 (2)	0.94717 (14)	0.0276 (6)	
H3	0.2913	0.7807	0.9850	0.033*	
C4	0.2167 (3)	0.6739 (2)	0.92216 (14)	0.0245 (6)	
H4	0.1416	0.6801	0.9450	0.029*	
C5	0.2229 (2)	0.5983 (2)	0.86127 (14)	0.0203 (6)	
C6	0.1319 (3)	0.6378 (3)	0.80715 (15)	0.0280 (7)	
H6A	0.0528	0.6448	0.8287	0.042*	
H6B	0.1278	0.5849	0.7692	0.042*	
H6C	0.1572	0.7091	0.7891	0.042*	
C7	0.3561 (2)	0.5809 (2)	0.83546 (14)	0.0190 (5)	
C8	0.3576 (3)	0.5283 (2)	0.76388 (14)	0.0233 (6)	
H8A	0.4396	0.5260	0.7443	0.028*	
H8B	0.3037	0.5670	0.7314	0.028*	
C9	0.3107 (3)	0.4125 (2)	0.78109 (14)	0.0244 (6)	
H9A	0.2310	0.3996	0.7601	0.029*	
H9B	0.3671	0.3555	0.7648	0.029*	
C10	0.3032 (2)	0.4144 (2)	0.86073 (13)	0.0184 (5)	
C11	0.1961 (2)	0.4800 (2)	0.88369 (13)	0.0186 (5)	
C12	0.1084 (3)	0.4403 (2)	0.92204 (14)	0.0227 (6)	
H12	0.0462	0.4885	0.9361	0.027*	
C13	0.1017 (3)	0.3215 (2)	0.94484 (14)	0.0233 (6)	
H13A	0.0202	0.2931	0.9356	0.028*	
H13B	0.1160	0.3173	0.9953	0.028*	
C14	0.1939 (3)	0.2494 (2)	0.90729 (13)	0.0209 (6)	
C15	0.1423 (3)	0.2191 (3)	0.83575 (15)	0.0272 (6)	
H15A	0.1188	0.2858	0.8112	0.041*	
H15B	0.0720	0.1720	0.8418	0.041*	
H15C	0.2033	0.1801	0.8089	0.041*	
C16	0.3147 (2)	0.3108 (2)	0.90233 (13)	0.0188 (6)	
H16	0.3370	0.3329	0.9504	0.023*	
C17	0.4044 (3)	0.2242 (2)	0.87993 (14)	0.0234 (6)	
H17A	0.4875	0.2476	0.8900	0.028*	
H17B	0.3971	0.2086	0.8299	0.028*	
C18	0.3695 (2)	0.1232 (2)	0.92347 (15)	0.0229 (6)	
H18	0.4193	0.1264	0.9664	0.027*	
C19	0.4019 (3)	0.0166 (2)	0.88754 (17)	0.0334 (7)	
H19A	0.3769	−0.0452	0.9164	0.050*	
H19B	0.4888	0.0135	0.8803	0.050*	
H19C	0.3609	0.0128	0.8428	0.050*	
C20	0.2361 (2)	0.1420 (2)	0.94671 (13)	0.0203 (6)	
C21	0.2739 (2)	0.0941 (2)	1.06562 (14)	0.0223 (6)	
C22	0.2662 (3)	0.1371 (3)	1.13866 (14)	0.0309 (7)	
H22A	0.1811	0.1396	1.1530	0.037*	
H22B	0.2979	0.2127	1.1399	0.037*	
C23	0.3360 (3)	0.0672 (3)	1.18969 (15)	0.0304 (7)	
H23A	0.3259	0.0967	1.2364	0.046*	
H23B	0.4212	0.0678	1.1773	0.046*	
H23C	0.3058	−0.0081	1.1882	0.046*	
C24	0.1462 (2)	0.0488 (2)	0.93501 (14)	0.0214 (6)	
C25	0.0457 (3)	0.0390 (3)	0.98795 (15)	0.0300 (7)	
H25A	0.0792	0.0143	1.0327	0.036*	
H25B	0.0082	0.1115	0.9951	0.036*	
C26	−0.1214 (3)	0.0001 (3)	0.91806 (15)	0.0277 (7)	
C27	−0.2012 (3)	−0.0907 (3)	0.89423 (15)	0.0295 (7)	
H27A	−0.2541	−0.0633	0.8570	0.035*	
H27B	−0.2526	−0.1145	0.9331	0.035*	
C28	−0.1304 (3)	−0.1883 (3)	0.86761 (19)	0.0401 (8)	
H28A	−0.1857	−0.2425	0.8482	0.060*	
H28B	−0.0853	−0.2212	0.9058	0.060*	
H28C	−0.0745	−0.1640	0.8316	0.060*	

### Atomic displacement parameters (Å^2^)

**Table:**

	*U*^11^	*U*^22^	*U*^33^	*U*^12^	*U*^13^	*U*^23^
Cl1	0.0214 (3)	0.0217 (4)	0.0479 (4)	−0.0017 (3)	0.0023 (3)	0.0073 (3)
Cl2	0.0363 (4)	0.0168 (3)	0.0244 (3)	−0.0009 (3)	−0.0006 (3)	0.0060 (3)
O1	0.0536 (14)	0.0287 (14)	0.0360 (11)	−0.0118 (11)	−0.0154 (11)	0.0019 (10)
O2	0.0217 (9)	0.0123 (9)	0.0219 (9)	−0.0012 (8)	0.0006 (8)	0.0013 (7)
O3	0.0273 (10)	0.0186 (10)	0.0162 (9)	−0.0005 (8)	0.0001 (7)	−0.0002 (7)
O4	0.0384 (11)	0.0173 (11)	0.0253 (10)	0.0010 (9)	0.0021 (9)	0.0009 (8)
O5	0.0447 (12)	0.0225 (12)	0.0260 (11)	−0.0040 (10)	0.0010 (9)	−0.0058 (9)
O6	0.0312 (11)	0.0301 (13)	0.0253 (10)	−0.0109 (9)	−0.0033 (9)	0.0064 (9)
O7	0.0477 (15)	0.0265 (13)	0.0581 (16)	0.0041 (11)	−0.0032 (11)	0.0130 (11)
C1	0.0258 (14)	0.0157 (14)	0.0234 (14)	0.0028 (11)	−0.0012 (11)	0.0039 (11)
C2	0.0393 (18)	0.0135 (14)	0.0214 (13)	−0.0032 (13)	−0.0081 (12)	0.0036 (11)
C3	0.0491 (18)	0.0150 (15)	0.0185 (13)	0.0053 (13)	0.0004 (13)	−0.0009 (11)
C4	0.0325 (15)	0.0195 (15)	0.0215 (13)	0.0060 (13)	0.0046 (12)	0.0033 (11)
C5	0.0200 (13)	0.0175 (15)	0.0234 (13)	−0.0016 (11)	0.0042 (11)	0.0027 (11)
C6	0.0304 (15)	0.0232 (16)	0.0305 (16)	0.0000 (13)	−0.0024 (12)	0.0066 (12)
C7	0.0224 (12)	0.0157 (14)	0.0188 (13)	0.0017 (11)	−0.0008 (10)	0.0008 (11)
C8	0.0315 (14)	0.0196 (15)	0.0188 (14)	0.0028 (12)	0.0041 (11)	0.0006 (11)
C9	0.0338 (15)	0.0176 (14)	0.0219 (14)	−0.0020 (12)	0.0002 (12)	0.0004 (12)
C10	0.0202 (13)	0.0153 (14)	0.0197 (12)	−0.0042 (11)	−0.0003 (10)	−0.0003 (10)
C11	0.0241 (13)	0.0131 (13)	0.0188 (13)	0.0007 (11)	−0.0041 (10)	−0.0002 (10)
C12	0.0233 (14)	0.0201 (14)	0.0247 (14)	0.0052 (12)	0.0002 (11)	−0.0004 (11)
C13	0.0218 (13)	0.0218 (15)	0.0262 (14)	−0.0003 (12)	0.0030 (12)	0.0018 (11)
C14	0.0248 (13)	0.0165 (15)	0.0213 (13)	−0.0029 (11)	0.0010 (11)	0.0013 (10)
C15	0.0338 (15)	0.0212 (15)	0.0267 (14)	−0.0063 (13)	−0.0049 (12)	0.0042 (12)
C16	0.0211 (12)	0.0167 (14)	0.0187 (13)	0.0004 (11)	0.0024 (10)	−0.0033 (10)
C17	0.0275 (13)	0.0155 (13)	0.0271 (13)	0.0013 (13)	0.0052 (12)	0.0017 (11)
C18	0.0272 (14)	0.0200 (15)	0.0214 (13)	0.0007 (12)	0.0023 (11)	0.0002 (11)
C19	0.0389 (16)	0.0170 (15)	0.0442 (18)	0.0054 (14)	0.0131 (15)	−0.0002 (13)
C20	0.0239 (13)	0.0189 (14)	0.0180 (12)	−0.0006 (11)	−0.0003 (10)	−0.0019 (11)
C21	0.0219 (13)	0.0233 (16)	0.0217 (13)	−0.0040 (12)	−0.0013 (10)	0.0007 (11)
C22	0.0422 (18)	0.0297 (18)	0.0209 (14)	0.0024 (15)	0.0012 (12)	−0.0021 (12)
C23	0.0331 (16)	0.0353 (19)	0.0228 (15)	−0.0038 (14)	−0.0012 (12)	0.0011 (13)
C24	0.0269 (14)	0.0166 (14)	0.0208 (14)	−0.0024 (12)	−0.0003 (11)	0.0014 (11)
C25	0.0292 (15)	0.0348 (19)	0.0259 (15)	−0.0137 (14)	0.0019 (12)	−0.0008 (13)
C26	0.0304 (16)	0.0291 (17)	0.0238 (14)	0.0015 (13)	0.0033 (12)	0.0048 (12)
C27	0.0272 (15)	0.0389 (19)	0.0224 (14)	−0.0047 (14)	−0.0033 (12)	0.0041 (13)
C28	0.046 (2)	0.0324 (19)	0.0417 (19)	−0.0022 (15)	−0.0091 (15)	−0.0061 (15)

### Geometric parameters (Å, °)

**Table:**

Cl1—C1	1.791 (3)	C13—H13A	0.9900
Cl2—C1	1.796 (3)	C13—H13B	0.9900
O1—C2	1.207 (3)	C14—C15	1.545 (4)
O2—C7	1.445 (3)	C14—C16	1.546 (4)
O2—C10	1.470 (3)	C14—C20	1.590 (4)
O3—C21	1.364 (3)	C15—H15A	0.9800
O3—C20	1.463 (3)	C15—H15B	0.9800
O4—C21	1.205 (4)	C15—H15C	0.9800
O5—C24	1.201 (3)	C16—C17	1.520 (4)
O6—C26	1.341 (3)	C16—H16	1.0000
O6—C25	1.428 (4)	C17—C18	1.545 (4)
O7—C26	1.193 (4)	C17—H17A	0.9900
C1—C7	1.514 (4)	C17—H17B	0.9900
C1—C2	1.548 (4)	C18—C19	1.521 (4)
C2—C3	1.458 (4)	C18—C20	1.572 (4)
C3—C4	1.332 (4)	C18—H18	1.0000
C3—H3	0.9500	C19—H19A	0.9800
C4—C5	1.500 (4)	C19—H19B	0.9800
C4—H4	0.9500	C19—H19C	0.9800
C5—C6	1.536 (4)	C20—C24	1.535 (4)
C5—C11	1.539 (4)	C21—C22	1.510 (4)
C5—C7	1.583 (4)	C22—C23	1.520 (4)
C6—H6A	0.9800	C22—H22A	0.9900
C6—H6B	0.9800	C22—H22B	0.9900
C6—H6C	0.9800	C23—H23A	0.9800
C7—C8	1.527 (4)	C23—H23B	0.9800
C8—C9	1.546 (4)	C23—H23C	0.9800
C8—H8A	0.9900	C24—C25	1.523 (4)
C8—H8B	0.9900	C25—H25A	0.9900
C9—C10	1.543 (4)	C25—H25B	0.9900
C9—H9A	0.9900	C26—C27	1.496 (4)
C9—H9B	0.9900	C27—C28	1.521 (5)
C10—C11	1.505 (4)	C27—H27A	0.9900
C10—C16	1.506 (4)	C27—H27B	0.9900
C11—C12	1.321 (4)	C28—H28A	0.9800
C12—C13	1.519 (4)	C28—H28B	0.9800
C12—H12	0.9500	C28—H28C	0.9800
C13—C14	1.538 (4)		
			
C7—O2—C10	96.57 (18)	H15A—C15—H15B	109.5
C21—O3—C20	117.1 (2)	C14—C15—H15C	109.5
C26—O6—C25	116.2 (3)	H15A—C15—H15C	109.5
C7—C1—C2	114.2 (2)	H15B—C15—H15C	109.5
C7—C1—Cl1	111.2 (2)	C10—C16—C17	119.3 (2)
C2—C1—Cl1	109.30 (19)	C10—C16—C14	111.5 (2)
C7—C1—Cl2	108.06 (19)	C17—C16—C14	104.7 (2)
C2—C1—Cl2	105.28 (19)	C10—C16—H16	106.9
Cl1—C1—Cl2	108.43 (15)	C17—C16—H16	106.9
O1—C2—C3	124.3 (3)	C14—C16—H16	106.9
O1—C2—C1	121.0 (3)	C16—C17—C18	103.7 (2)
C3—C2—C1	114.7 (2)	C16—C17—H17A	111.0
C4—C3—C2	123.6 (3)	C18—C17—H17A	111.0
C4—C3—H3	118.2	C16—C17—H17B	111.0
C2—C3—H3	118.2	C18—C17—H17B	111.0
C3—C4—C5	125.9 (3)	H17A—C17—H17B	109.0
C3—C4—H4	117.0	C19—C18—C17	112.1 (2)
C5—C4—H4	117.0	C19—C18—C20	118.7 (3)
C4—C5—C6	108.1 (2)	C17—C18—C20	106.1 (2)
C4—C5—C11	110.5 (2)	C19—C18—H18	106.4
C6—C5—C11	111.1 (2)	C17—C18—H18	106.4
C4—C5—C7	111.9 (2)	C20—C18—H18	106.4
C6—C5—C7	116.6 (2)	C18—C19—H19A	109.5
C11—C5—C7	98.3 (2)	C18—C19—H19B	109.5
C5—C6—H6A	109.5	H19A—C19—H19B	109.5
C5—C6—H6B	109.5	C18—C19—H19C	109.5
H6A—C6—H6B	109.5	H19A—C19—H19C	109.5
C5—C6—H6C	109.5	H19B—C19—H19C	109.5
H6A—C6—H6C	109.5	O3—C20—C24	108.3 (2)
H6B—C6—H6C	109.5	O3—C20—C18	109.1 (2)
O2—C7—C1	106.9 (2)	C24—C20—C18	117.9 (2)
O2—C7—C8	102.0 (2)	O3—C20—C14	105.1 (2)
C1—C7—C8	117.1 (2)	C24—C20—C14	110.5 (2)
O2—C7—C5	102.8 (2)	C18—C20—C14	105.3 (2)
C1—C7—C5	115.3 (2)	O4—C21—O3	123.2 (2)
C8—C7—C5	110.7 (2)	O4—C21—C22	126.4 (3)
C7—C8—C9	100.7 (2)	O3—C21—C22	110.3 (2)
C7—C8—H8A	111.6	C21—C22—C23	112.5 (3)
C9—C8—H8A	111.6	C21—C22—H22A	109.1
C7—C8—H8B	111.6	C23—C22—H22A	109.1
C9—C8—H8B	111.6	C21—C22—H22B	109.1
H8A—C8—H8B	109.4	C23—C22—H22B	109.1
C10—C9—C8	102.7 (2)	H22A—C22—H22B	107.8
C10—C9—H9A	111.2	C22—C23—H23A	109.5
C8—C9—H9A	111.2	C22—C23—H23B	109.5
C10—C9—H9B	111.2	H23A—C23—H23B	109.5
C8—C9—H9B	111.2	C22—C23—H23C	109.5
H9A—C9—H9B	109.1	H23A—C23—H23C	109.5
O2—C10—C11	98.9 (2)	H23B—C23—H23C	109.5
O2—C10—C16	111.0 (2)	O5—C24—C25	121.1 (3)
C11—C10—C16	111.0 (2)	O5—C24—C20	122.6 (3)
O2—C10—C9	102.0 (2)	C25—C24—C20	116.1 (2)
C11—C10—C9	110.2 (2)	O6—C25—C24	110.6 (2)
C16—C10—C9	121.1 (2)	O6—C25—H25A	109.5
C12—C11—C10	123.9 (3)	C24—C25—H25A	109.5
C12—C11—C5	130.3 (3)	O6—C25—H25B	109.5
C10—C11—C5	105.3 (2)	C24—C25—H25B	109.5
C11—C12—C13	123.4 (3)	H25A—C25—H25B	108.1
C11—C12—H12	118.3	O7—C26—O6	123.6 (3)
C13—C12—H12	118.3	O7—C26—C27	126.2 (3)
C12—C13—C14	112.3 (2)	O6—C26—C27	110.2 (3)
C12—C13—H13A	109.2	C26—C27—C28	112.2 (3)
C14—C13—H13A	109.2	C26—C27—H27A	109.2
C12—C13—H13B	109.2	C28—C27—H27A	109.2
C14—C13—H13B	109.2	C26—C27—H27B	109.2
H13A—C13—H13B	107.9	C28—C27—H27B	109.2
C13—C14—C15	108.2 (2)	H27A—C27—H27B	107.9
C13—C14—C16	109.5 (2)	C27—C28—H28A	109.5
C15—C14—C16	112.7 (2)	C27—C28—H28B	109.5
C13—C14—C20	116.4 (2)	H28A—C28—H28B	109.5
C15—C14—C20	110.0 (2)	C27—C28—H28C	109.5
C16—C14—C20	100.0 (2)	H28A—C28—H28C	109.5
C14—C15—H15A	109.5	H28B—C28—H28C	109.5
C14—C15—H15B	109.5		
			
C7—C1—C2—O1	145.1 (3)	C5—C11—C12—C13	−174.1 (3)
Cl1—C1—C2—O1	19.8 (3)	C11—C12—C13—C14	−10.9 (4)
Cl2—C1—C2—O1	−96.5 (3)	C12—C13—C14—C15	−81.5 (3)
C7—C1—C2—C3	−36.6 (3)	C12—C13—C14—C16	41.7 (3)
Cl1—C1—C2—C3	−162.0 (2)	C12—C13—C14—C20	154.1 (2)
Cl2—C1—C2—C3	81.7 (3)	O2—C10—C16—C17	−80.9 (3)
O1—C2—C3—C4	−165.8 (3)	C11—C10—C16—C17	170.1 (2)
C1—C2—C3—C4	16.0 (4)	C9—C10—C16—C17	38.5 (4)
C2—C3—C4—C5	−2.5 (5)	O2—C10—C16—C14	156.8 (2)
C3—C4—C5—C6	−120.7 (3)	C11—C10—C16—C14	47.9 (3)
C3—C4—C5—C11	117.5 (3)	C9—C10—C16—C14	−83.7 (3)
C3—C4—C5—C7	9.1 (4)	C13—C14—C16—C10	−62.1 (3)
C10—O2—C7—C1	177.9 (2)	C15—C14—C16—C10	58.3 (3)
C10—O2—C7—C8	−58.6 (2)	C20—C14—C16—C10	175.1 (2)
C10—O2—C7—C5	56.1 (2)	C13—C14—C16—C17	167.5 (2)
C2—C1—C7—O2	−69.4 (3)	C15—C14—C16—C17	−72.0 (3)
Cl1—C1—C7—O2	54.9 (2)	C20—C14—C16—C17	44.7 (2)
Cl2—C1—C7—O2	173.80 (16)	C10—C16—C17—C18	−167.4 (2)
C2—C1—C7—C8	177.0 (2)	C14—C16—C17—C18	−41.8 (3)
Cl1—C1—C7—C8	−58.7 (3)	C16—C17—C18—C19	152.0 (2)
Cl2—C1—C7—C8	60.2 (3)	C16—C17—C18—C20	20.9 (3)
C2—C1—C7—C5	44.1 (3)	C21—O3—C20—C24	65.7 (3)
Cl1—C1—C7—C5	168.45 (18)	C21—O3—C20—C18	−63.7 (3)
Cl2—C1—C7—C5	−72.6 (2)	C21—O3—C20—C14	−176.2 (2)
C4—C5—C7—O2	86.0 (3)	C19—C18—C20—O3	126.9 (3)
C6—C5—C7—O2	−148.8 (2)	C17—C18—C20—O3	−105.9 (2)
C11—C5—C7—O2	−30.1 (2)	C19—C18—C20—C24	3.0 (4)
C4—C5—C7—C1	−29.9 (3)	C17—C18—C20—C24	130.2 (3)
C6—C5—C7—C1	95.3 (3)	C19—C18—C20—C14	−120.7 (3)
C11—C5—C7—C1	−146.0 (2)	C17—C18—C20—C14	6.5 (3)
C4—C5—C7—C8	−165.7 (2)	C13—C14—C20—O3	−33.2 (3)
C6—C5—C7—C8	−40.5 (3)	C15—C14—C20—O3	−156.7 (2)
C11—C5—C7—C8	78.2 (2)	C16—C14—C20—O3	84.6 (2)
O2—C7—C8—C9	39.8 (2)	C13—C14—C20—C24	83.4 (3)
C1—C7—C8—C9	156.1 (2)	C15—C14—C20—C24	−40.1 (3)
C5—C7—C8—C9	−69.1 (3)	C16—C14—C20—C24	−158.8 (2)
C7—C8—C9—C10	−5.8 (3)	C13—C14—C20—C18	−148.3 (2)
C7—O2—C10—C11	−59.3 (2)	C15—C14—C20—C18	88.2 (3)
C7—O2—C10—C16	−175.9 (2)	C16—C14—C20—C18	−30.6 (2)
C7—O2—C10—C9	53.8 (2)	C20—O3—C21—O4	−2.9 (4)
C8—C9—C10—O2	−29.0 (3)	C20—O3—C21—C22	179.8 (2)
C8—C9—C10—C11	75.3 (3)	O4—C21—C22—C23	16.6 (4)
C8—C9—C10—C16	−152.8 (2)	O3—C21—C22—C23	−166.2 (2)
O2—C10—C11—C12	−132.5 (3)	O3—C20—C24—O5	−161.5 (3)
C16—C10—C11—C12	−15.8 (4)	C18—C20—C24—O5	−37.1 (4)
C9—C10—C11—C12	121.2 (3)	C14—C20—C24—O5	83.9 (3)
O2—C10—C11—C5	40.5 (2)	O3—C20—C24—C25	23.5 (3)
C16—C10—C11—C5	157.1 (2)	C18—C20—C24—C25	147.9 (3)
C9—C10—C11—C5	−65.9 (3)	C14—C20—C24—C25	−91.1 (3)
C4—C5—C11—C12	48.3 (4)	C26—O6—C25—C24	−79.3 (3)
C6—C5—C11—C12	−71.6 (4)	O5—C24—C25—O6	−5.4 (4)
C7—C5—C11—C12	165.6 (3)	C20—C24—C25—O6	169.7 (2)
C4—C5—C11—C10	−124.0 (2)	C25—O6—C26—O7	−4.1 (4)
C6—C5—C11—C10	116.1 (2)	C25—O6—C26—C27	174.9 (2)
C7—C5—C11—C10	−6.7 (2)	O7—C26—C27—C28	124.6 (4)
C10—C11—C12—C13	−3.1 (4)	O6—C26—C27—C28	−54.3 (3)

## References

[bb1] Barbour, L. J. (2001). *J. Supramol. Chem.* **1**, 189–191.

[bb2] Bruker (2008). *APEX2* and *SAINT* Bruker AXS Inc., Madison, Wisconsin, USA.

[bb3] Flack, H. D. (1983). *Acta Cryst.* A**39**, 876–881.

[bb4] Ketuly, K. A. A., Hadi, A. H. & Ng, S. W. (2009*a*). *Acta Cryst.* E**65**, o1821. 10.1107/S1600536809025975 PMC2977184 21583523

[bb5] Ketuly, K. A., Hadi, A. H. A. & Ng, S. W. (2009*b*). *Acta Cryst.* E**65**, o1822. 10.1107/S1600536809025987 PMC2977202 21583524

[bb6] Sheldrick, G. M. (1996). *SADABS.* University of Göttingen, Germany.

[bb7] Sheldrick, G. M. (2008). *Acta Cryst.* A**64**, 112–122. 10.1107/S0108767307043930 18156677

[bb8] Westrip, S. P. (2009). *publCIF* In preparation.

